# Optimizing contact tracing for avian influenza in poultry flocks

**DOI:** 10.1098/rsif.2024.0523

**Published:** 2025-01-08

**Authors:** Sébastien Lambert, Lisa Fourtune, Peter H. F. Hobbelen, Julie Baca, José L. Gonzales, Armin R. W. Elbers, Timothée Vergne

**Affiliations:** ^1^ IHAP, Université de Toulouse, INRAE, ENVT, Toulouse, France; ^2^ Department of Epidemiology, Bioinformatics and Animal Models, Wageningen Bioveterinary Research, Lelystad, The Netherlands

**Keywords:** mechanistic modelling, time of first infection, highly pathogenic avian influenza, approximate Bayesian computation, mortality data, Shiny app

## Abstract

Contact tracing is commonly used to manage infectious diseases of both humans and animals. It aims to detect early and control potentially infected individuals or farms that had contact with infectious cases. Because it is very resource-intensive, contact tracing is usually performed on a pre-defined time window, based on previous knowledge of the duration of the incubation period. However, pre-defined time windows may not be always relevant, reducing the efficiency of contact tracing. In this study, we estimated the day when farms were first infected with highly pathogenic avian influenza viruses, a devastating pathogen causing severe socio-economic damage in domestic poultry. The estimation was performed by fitting a stochastic mechanistic model to observed daily mortality data from 63 infected poultry farms in France and The Netherlands, using approximate Bayesian computation. Independent of the poultry species or country, the estimates of the time of first infection ranged between 3.4 (95% credible interval—CrI: 2.6, 4.6) and 19.9 (95% CrI: 11.9, 31.3) days prior to the last observation. We developed an online application to provide real-time support to policymakers by estimating realistic ranges of dates of first infection to inform contact tracing and improve its efficiency.

## 1. Introduction

Contact tracing belongs to the basic toolbox to manage infectious diseases. Its aim is to target surveillance or intervention measures (such as quarantine, vector control or depopulation) towards epidemiological units that are at higher risk of infection because of their contact with an infected unit [1–3]. Focusing control measures on high-risk epidemiological units has been shown to improve their impact on the spread of infectious diseases [4–7]. Indeed, targeted surveillance can improve the timeliness of detection of recent infections, quarantine or vector control can decrease the contact rate for still-susceptible units and depopulation can substantially reduce the duration of the infectious period. However, since it is extremely time-consuming, the ability to trace and manage a sufficient number of contacts depends on the availability of operational and logistical resources [8], and also on the characteristics of the disease itself that influence the time between the first infection and detection [9–11]. Contact tracing remains especially useful when the number of infectious cases is low, i.e. for (re-)emerging infectious diseases (EIDs) [9]. As such, it has been an integral part of strategies against both human (e.g. SARS, Ebola or COVID-19) and animal (e.g. foot-and-mouth disease (FMD), classical swine fever) EIDs [2,12–16] and is likely to remain so in the future.

For infectious diseases of livestock, contact tracing is performed by official veterinarians to identify the potential origin of the outbreak (backward tracing) as well as potential secondary cases (forward tracing) [15,17,18]. Depending on the disease transmission routes, relevant contacts that need to be traced include the movement of animals, persons, products, vehicles and material to, from or through the outbreak farm. To achieve this, investigators use various methods and tools, such as interviews of farmers, examination of farm logbooks, investigation of transport records and analysis of official movement databases [15]. Measures that are applied in the at-risk farms identified through contact tracing include: clinical and laboratory examinations, movement bans (on animals, products and materials), isolation of animals, strengthening of biosecurity measures and potentially culling [18]. For instance, during the 2001 FMD epidemic in the UK, premises where animals had been in direct contact with infected animals or were exposed to infection by any other transmission route were preventively culled [1].

Estimating the probable range of date of first infection in a farm would help tracing activities by enabling official veterinarians to focus on a precise and focused list of potential sources of infection and on relevant contacts from the infected farm that happened during the infectious period. However, such estimates of the date of first infection are usually not available when an outbreak is detected [15]. Because of logistical and resource constraints, contact tracing is therefore usually performed on a pre-defined time window, which is often based on the duration of the incubation period at the individual level [19]. For instance, the ‘Animal Health Law’ in the European Union defines a minimal time window of 15 days for classical and African swine fever and 21 days for FMD and highly pathogenic avian influenza (HPAI) [17].

Despite these time windows being disease specific, they may not be adequate for each outbreak because the within-farm transmission dynamics may depend on the pathogen (e.g. different strains) and farm-specific factors. Moreover, the timeliness of suspicion reporting also depends on the farmers’ disease awareness. Consequently, the delay between the first infection and the notification of the suspicion could be shorter than the time window, meaning that a lot of effort and resources are used to trace contacts that happened before probable infection dates and are therefore not relevant for surveillance and control. Alternatively, the delay could be longer, meaning that epidemiological links, including the potential source of infection, could be missed by contact tracing when sticking to the pre-defined time window. As contact tracing is very resource-demanding, focusing efforts on the most relevant period through the estimation of the farm-specific date of first infection could help increase efficiency. This can be done by reconstructing within-farm transmission dynamics using epidemiological models fitted to relevant data collected on farms (such as results of diagnostic tests, production drops, mortality, etc.) [20–27].

Using HPAI as a case study, our aim was twofold: (i) to estimate outbreak-specific most likely dates of first infection using a mechanistic model and approximate Bayesian computation in various settings (e.g. various poultry species, various HPAI subtypes) and (ii) to make this modelling tool available for direct use by official veterinarians through an online application, with the applied objective of optimizing contact tracing during future epidemics. Previous studies were able to retrospectively estimate dates of first infection using daily mortality data from a limited number of HPAI-infected flocks (*n* = 8 in [20], *n* = 1 in [21], *n* = 7 in [22] and *n* = 12 in [23]). In our study, daily mortality data from 63 HPAI-infected flocks were included, representing the most comprehensive dataset analysed so far.

## 2. Methods

### 2.1. Data

We analysed daily mortality data from layer, broiler and breeder chicken (*n* = 27), broiler Pekin duck (*n* = 10) and broiler turkey (*n* = 4) flocks gathered by The Netherlands Food and Consumer Product Safety Authority (NVWA) from 41 Dutch outbreaks of clade 2.3.4.4 HPAI viruses during the years 2014–15 (H5N8, *n* = 5), 2016–17 (H5N8, *n* = 7), 2017–2018 (H5N6, *n* = 3), 2020–21 (H5N8, *n* = 11) and 2021–22 (H5N1, *n* = 15). We also analysed daily mortality data from 11 layer, broiler and breeder chicken and 11 mule duck outbreaks in France during the years 2016–17 (H5N8, *n* = 17), 2020–21 (H5N8, *n* = 1) and 2021–22 (H5N1, *n* = 4). Irrespective of the country, the mean flock size (number of birds) was 20 011 chickens (minimum: 4200, maximum: 63 540), 6965 turkeys (min.: 4350, max.: 12 240), 11 001 Pekin ducks (min.: 7800, max.: 15 000), and 4847 mule ducks (min.: 945, max: 10 506).

We focused on the initial exponential increase in mortality due to HPAI. For six flocks, mortality decreased during the last 4–6 days, but it was highly suspected that this decrease was due to an underreporting by the farmer (e.g. when the number of dead birds became too high to count or after the HPAI infection was confirmed). Therefore, the last observation for each flock was the day with the highest mortality incidence (hereafter referred to as ‘the last observation’).

For all flocks, we kept at most 35 days (5 weeks) of daily mortality data before the last observation. Across this 5 week period, we ignored the daily mortality incidence related to the first week of life in nine broiler duck flocks, since high daily mortality rates due to causes other than HPAI infections are often observed during that period [28]. For the same reason, we did not use the mortality data of the first 10 days of life in six chicken broiler flocks [29]. In six flocks, we also ignored abnormal mortality events (from 1–9 days) above pre-defined thresholds [28–31] that were observed during the 35 days prior to the last observation, except for the event that led to the detection of HPAI infection. Overall, an average of eight data points (min: 1–max: 16) were removed in the five weeks preceding the last observation in 19 flocks.

Finally, an average of 27 data points were used for each flock (min: 3–max: 36; see electronic supplementary material).

### 2.2. Mechanistic model

**Figure 1.**
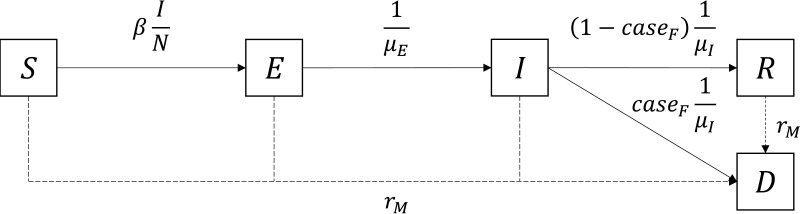
Compartmental model used to estimate the within-flock transmission dynamics of highly pathogenic avian influenza viruses. S: susceptible; E: exposed (infected but not yet infectious); I: infectious; R: recovered; D: deceased; N: total number of live birds; β: transmission rate (per day); $\mu_{E}$: average duration of the latent period (days); $\mu_{I}$: average duration of the infectious period (days); ${case}_{F}$: probability of dying from infection; $r_{M}$: baseline mortality rate (per day).

To estimate the time of first infection from daily mortality data, we adapted a previously developed modelling framework that was first applied to within-herd dynamics of African swine fever [24] and then to within-flock dynamics of HPAI [21]. Briefly, we used a stochastic SEIRD compartmental model (figure 1) to describe the transmission dynamics before the whole flock was depopulated. Individual birds were divided into five compartments: susceptible (S), exposed (infected but not yet infectious, E), infectious (I), recovered (R) and deceased (D). The output of interest was the number of dead birds (either from baseline mortality or as a result of HPAI infection) each day. We considered a single homogeneously mixing population within a poultry house and a frequency-dependent contact rate [21]. Therefore, the force of infection in the model was given by


$$\lambda \left(t\right)=\beta \frac{I\left(t\right)}{N\left(t\right)},$$

where β is the transmission rate (the number of individuals effectively contacted by each individual per unit time), I(t) is the number of infectious birds at time t and N(t) is the total number of live birds at time t: N(t)=S(t)+E(t)+I(t)+R(t). At the initial time step, $N(t)=S(t)=N_{0}$, the initial number of (susceptible) birds in the flock. The first infection was assumed to happen at $t=t_{0}$ (time of first infection) by moving one susceptible (S) individual into the exposed (E) compartment [21]. The durations of the latent and infectious periods were assumed to follow gamma distributions with means $\mu_{E}$ and $\mu_{I}$ and shape $k_{E}$ and $k_{I}$, respectively [32]. However, a sensitivity analysis showed that the shape parameters $k_{E}$ and $k_{I}$ were not influential on the daily number of dead birds (see electronic supplementary material). Thus, for the sake of simplicity, we fixed $k_{E}=k_{I}=1$, i.e. assuming exponential distributions for the duration of the latent and infectious periods. At the end of the infectious period, birds could either die (D) or survive the infection and recover (R), according to the case fatality risk $case_{F}$ [21]. Birds from all compartments could also die from other causes (i.e. other than HPAI), according to the per capita baseline mortality rate $r_{M}$ [21]. See the electronic supplementary material for more details on the model.

### 2.3. Parameter estimation

The transmission rate β, the average durations of the latent and infectious periods ($\mu_{E}$ and $\mu_{I}$), the probability of dying from infection ${case}_{F}$, the per capita baseline mortality rate $r_{M}$ and the time of first infection $t_{0}$ were estimated for each flock independently. Parameters were estimated using approximate Bayesian computation sequential Monte Carlo (ABC-SMC) methods [21,24,33]. More precisely, we used the algorithm of Lenormand *et al*. [34] implemented in the R package EasyABC [35,36]. Briefly, several SMC steps were performed sequentially. At each step, parameter sets (also called ‘particles’) were sampled. Then, the model was used to simulate daily mortality incidence for each particle. Finally, the particles were kept if the Euclidean distance between simulated and observed daily mortality incidence was less than a threshold defined at the previous SMC step. At each step, the distance threshold decreased. Initial parameter sets (*n* = 10 000) were sampled from the prior distributions, while for subsequent SMC steps, particles were sampled from the particles kept at the previous round with a perturbation kernel (Gaussian kernel with twice the weighted empirical variance of the previous sample [34]). The algorithm stopped when resampling new particles stopped improving the model fit to observed data. The 5000 accepted parameter sets from the final SMC step approximated the posterior distribution. The algorithm is described in detail in the electronic supplementary material.

Prior distributions were constructed based on a thorough literature review, with some prior distributions being species specific while others were not (table 1 and electronic supplementary material). To explore the sensitivity of the results to the prior distributions, parameter estimation was also performed after having changed the informative prior distributions of β, $\mu_{I}$ and ${case}_{F}$ to uniform distributions with biologically realistic ranges. More details are available in the electronic supplementary material. We assessed how much the data contributed to the posterior distributions by calculating the overlap (range: 0–1) between each marginal prior–posterior pair, with a low overlap indicative of a strong identifiability [44]. We also examined the correlations between each pair of parameters (see electronic supplementary material). Finally, we performed posterior predictive checks by visually comparing simulated and observed daily mortality incidence. Simulated daily mortality incidence was obtained by running 12 000 iterations of the model, sampling parameter values from the joint posterior distributions. The model fit was deemed satisfactory when at least 50% of the observed daily mortality during the exponential increase lied within the 50% projection intervals.

**Table 1.** Prior distributions used to estimate within-flock transmission parameters for highly pathogenic avian influenza in France and The Netherlands.

parameter (dimension)	prior distributions	references^a^
$t_{0}$: time of first infection (day)	$U\left(max\left(-35,t_{start}\right),0\right)$ ^b^	[23]
β: transmission rate (per day)	*PERT* (0, 1.6, 35)^c^	[37,38]
$\mu_{E}$: average duration of the latent period (day)	$U\left(0,3\right)$	[39]
$\mu_{I}$: average duration of the infectious period (day)	C,T: gamma(5.34, 0.61)^d^	[40]
	D: gamma(5.16, 1.63)^e^	
${case}_{F}$: probability of dying of disease	C,T: $PERT\left(0,1,1\right)$^c^^,^^d^	[20,21,41–43]
	MD: PERT(0, 0.795, 1)^c^^,f^	
	PD: PERT(0, 0.125, 1)^c^^,g^	
$r_{M}$: baseline mortality rate (per day)	$U\left(0,0.003\right)$	[28]

^a^ See details in the electronic supplementary material.

^b^ $t_{start}$ is the start of the production cycle.

^c^ PERT distribution of parameters (minimum, mode, maximum).

^d^ C: chickens/T: turkeys.

^e^ D: ducks.

^f^ MD: mule ducks (sterile cross-breeds between male Muscovy ducks *Cairina moschata* and female Pekin ducks *Anas platyrhynchos domesticus*).

^g^ PD: Pekin ducks (*Anas platyrhynchos domesticus*).

All the analyses were performed using R statistical software [45].

### 2.4. Alternative approach for the Shiny app

Because ABC-SMC uses a sequential approach, the fitting algorithm requires new simulations to be run for every flock, which takes several hours. Therefore, this approach was not deemed suitable for providing results quickly within an online application. To decrease the computational time from several hours to a few minutes, we tested whether ABC rejection [46,47] could be used instead of ABC-SMC. Indeed, although ABC-SMC is more efficient, ABC rejection has the advantage that model simulations can be run first as they are not sequential. With a slight modification (using daily proportion instead of daily number of dead birds as summary statistics) allowing independence from the initial flock size (see electronic supplementary material), we were thus able to run in advance all the model simulations, sampling 5 000 000 parameter values from the prior distributions. We had three sets of ready-to-use model simulations because of species-dependent prior distributions (i.e. one for chickens and turkeys, one for Pekin ducks and one for mule ducks) (table 1). We compared the estimates of the time of first infection between ABC-SMC and ABC rejection by comparing the posterior distributions visually and by calculating the overlap between each posterior–posterior pair.

## 3. Results

**Figure 2.**
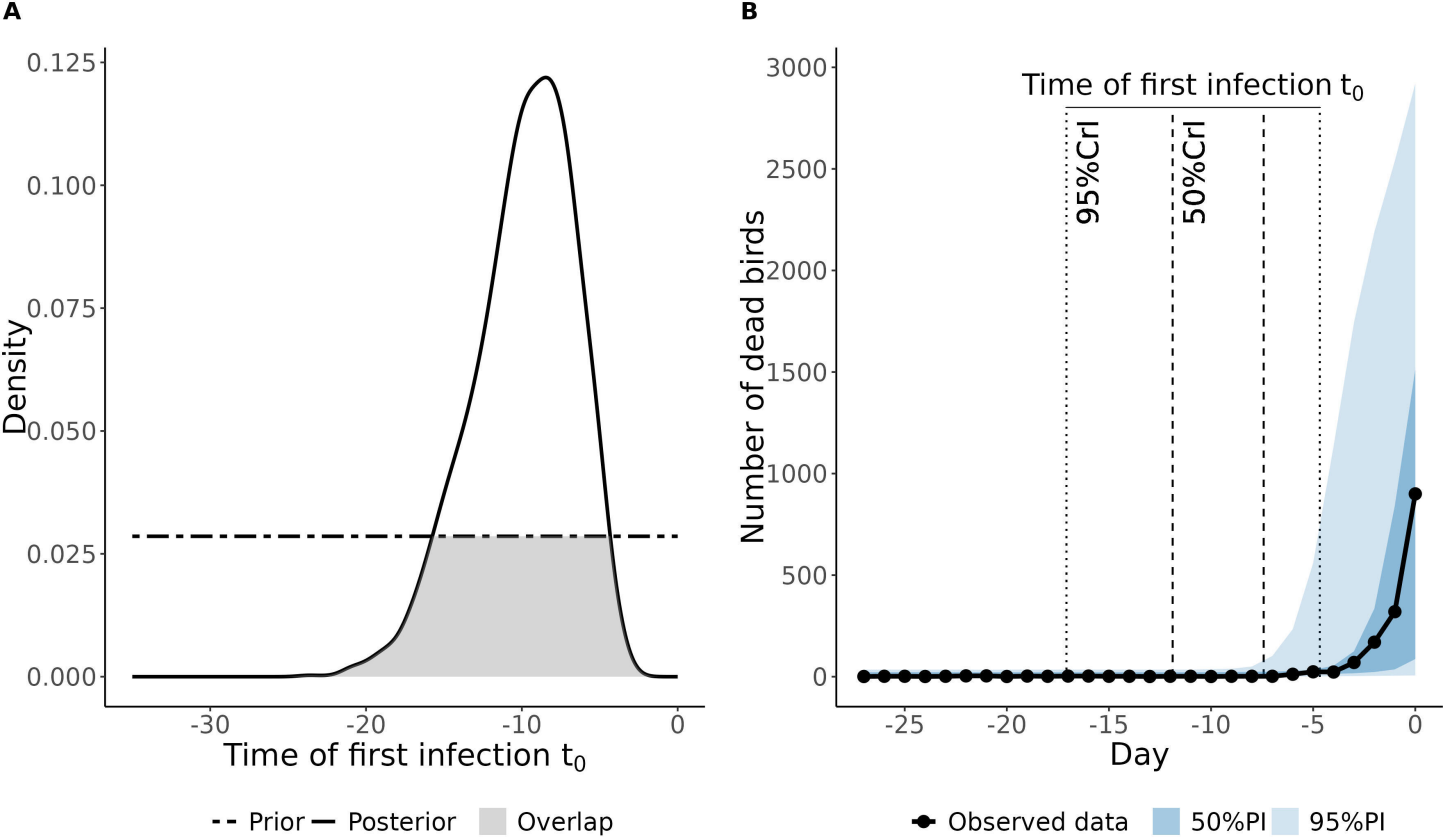
Posterior distribution of the time of first infection and model fit to observed data for the index case of the 2014–15 HPAI H5N8 epidemic in The Netherlands. (*a*) Prior (dashed line) and posterior (solid line) distributions of the time of first infection, with overlap between the two distributions (grey area). (*b*) Daily number of dead birds over time, with day 0 being the day of the last observation included in the model; the dark and light blue areas represent respectively the 50% and 95% projection intervals (PI); the black dots and solid lines represent the observed daily mortality data; the vertical dotted and dashed lines represent respectively the 50% and 95% credible intervals (CrI) of the time of first infection.

The posterior distributions of the six parameters were estimated for each flock independently. As an illustration, we showed the predicted daily mortality for the index case of the 2014–15 HPAI H5N8 epidemic in The Netherlands (figure 2). For this specific flock, the time of first infection was estimated to happen between 4.7 and 17.1 days before the last observation, as illustrated in figure 2*a*. The overlap between the prior and posterior distribution was 0.40. The model adequately captured the trend in mortality, with the observed daily mortality lying close to the centre of the prediction intervals (figure 2*b*). More details for this specific example are available in the electronic supplementary material.

**Figure 3.**
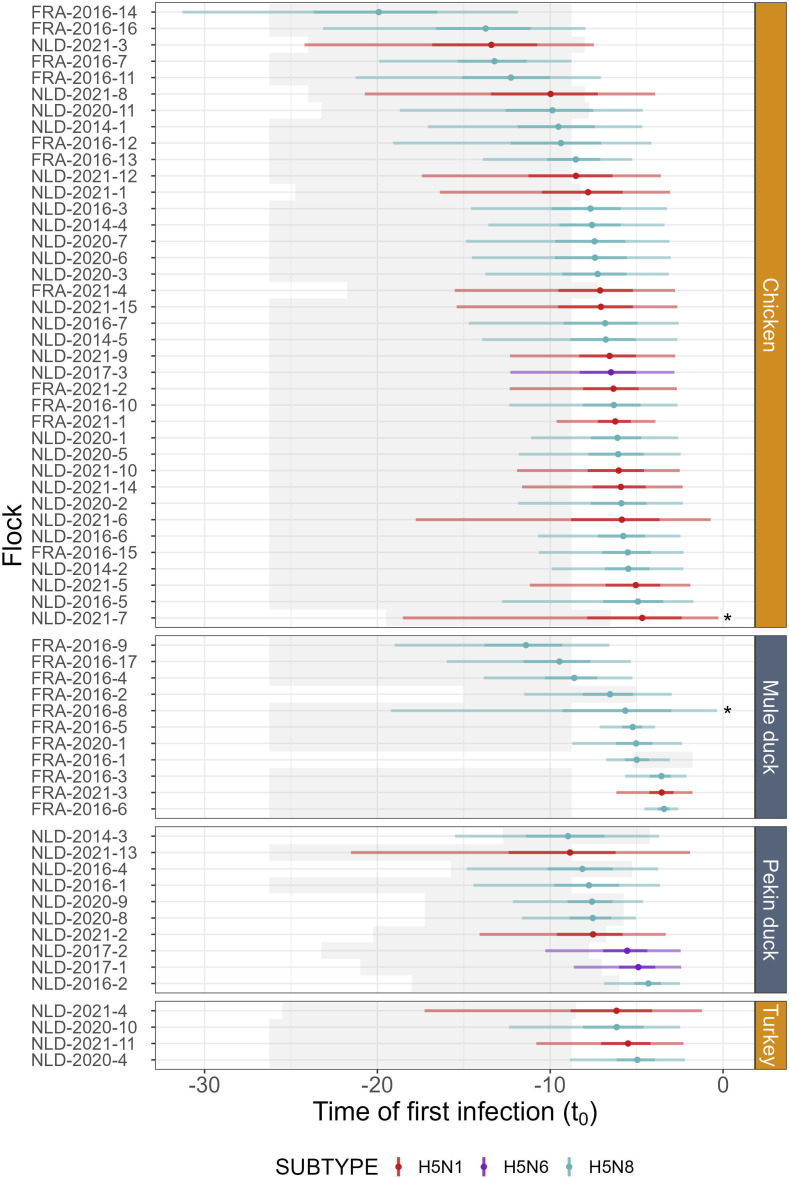
Posterior distributions of the flock-specific times of first infection. Times of first infection were estimated for each flock separately. Results are presented in the ascending order of the median $t_{0}$ for each species (chickens, mule ducks, pekin ducks and turkeys). Colours indicate virus subtypes (H5N1, H5N6 and H5N8). Dots indicate the median of the posterior distribution, with 95% and 50% credible intervals indicated by light- and dark-shaded bars, respectively. For comparison, the grey rectangles in the background represent the interquartile range of the prior distributions for each flock. The prior followed a uniform distribution between −35 and 0, except for flocks where the production cycle started (i.e. animals were introduced into the poultry house) less than 35 days ago. The two flocks presenting a bad fit to the observed data in the posterior predictive checks are indicated by a star.

The estimates of the time of first infection for each flock ranged between a median of 4.7 (95% equal-tailed credible interval—CrI: 0.27, 18.5) and 19.9 (95% CrI: 11.9, 31.3) days prior to the last observation in chickens, between 5.0 (95% CrI: 2.2, 8.8) and 6.2 (95% CrI: 1.2, 17.3) days in turkeys, between 3.4 (95% CrI: 2.6, 4.6) and 11.4 (95% CrI: 6.6, 19.0) days in mule ducks, and between 4.3 (95% CrI: 2.5, 6.9) and 9.0 (95% CrI: 3.7, 15.5) days in Pekin ducks (figure 3 and electronic supplementary material). The overlap between each marginal prior–posterior pair (one for each flock) for this parameter ranged between 0.08 and 0.69, with 50% of the flocks having overlaps between 0.30 and 0.44, indicating that the data supplied information about this parameter.

The flock-specific estimates of the other five parameters are provided in the electronic supplementary material. Except for the baseline mortality rate, the prior–posterior overlaps of these parameters were really high for most flocks, indicating that the data were rarely informative for these parameters.

The posterior predictive checks showed that the model adequately captured the trend in mortality for 61 out of 63 flocks, but not for two flocks that presented a bad fit to the observed data. For these two flocks, the upper bound of the 95% CrI of the time of first infection was equal to or greater than 0.5 days prior to the last observation, which was not the case for any of the flocks presenting a good fit (electronic supplementary material). The choice of prior distributions did not have a substantial influence on the results (see electronic supplementary material). These results suggest that efficient modelling pipelines fed with daily mortality data in outbreak farms could provide real-time support to policymakers by estimating probable ranges of dates of first infections to inform contact tracing. Therefore, we wanted to develop an online application to allow veterinary services to apply this modelling tool during future epidemics.

However, ABC-SMC needs too much running time to be used efficiently in an online application. Thus, we compared the results of ABC-SMC with an alternative method, ABC rejection, for which pre-made simulations stored within the application could be used. With a few exceptions, the results were consistent between ABC-SMC and ABC rejection for the estimates of the time of first infection (ranging between 3.4 (95% CrI: 1.5, 7.0) and 18.9 (95% CrI: 8.9, 33.5) days prior to the last observation), and the model fit was good for most flocks (60 out of 63). See the electronic supplementary material for more details.

Based on these results, we created an interactive application using the R package Shiny [48] that can be accessed online at: https://first-inf.sk8.inrae.fr/. Our Shiny app consists of three different tabs. When the application is loaded, the first tab introduces the context in which the app was developed, presents its objective and describes how to use the app. Within the second tab, the user is invited to enter the required data, i.e. the poultry species, the initial flock size, the date at which the flock was introduced in the poultry house and the date of the last observation (last available daily mortality incidence). Based on the latter, a table is generated to enter the daily mortality data. The table spans between the date of the last observation and the date of flock introduction, with an upper limit of 5 weeks before the last observation. The user is then invited to enter the daily mortality incidence for as many days as possible (by default the value is ‘−1’, corresponding to missing values). When all the data are entered and validated by the user, the third tab is displayed. A ‘start simulation’ button is displayed at the top of the page. When clicked, the ABC rejection algorithm is first performed, and subsequently 1000 iterations of the model are simulated, sampling parameter values from the joint posterior distributions. The estimates of the dates of first infection are shown and the simulations are used to visually compare simulated and observed daily mortality incidence in a plot similar to figure 2*b*. Based on the results, recommendations for optimizing contact tracing are formulated, except when the estimation is suspected to have failed (e.g. very high overlap or bad fit of the model to observed data), in which case warnings are displayed.

## 4. Discussion

We estimated flock-specific times of first infection for 63 HPAI-infected chicken, turkey and duck flocks from France and The Netherlands. To estimate those parameters, we used a mechanistic SEIRD model that was fitted to daily mortality data using approximate Bayesian computation. Although median estimates were all consistent with the current 21 day window, we found high variability in the estimates of the time of first infection, which ranged between 3.4 (95% CrI: 2.6, 4.6) and 19.9 (95% CrI: 11.9, 31.3) days prior to the last observation included in the model (figure 3). Such variability in the time of first infection between flocks may be explained by differences between flocks (host species, production type, country) and viruses (subtype, strain), which will influence within-flock transmission dynamics. However, it may also vary depending on farmers’ awareness, which will influence the timeliness in detecting clinical signs or abnormal mortality and subsequently reporting a suspicion. In the future, it would be interesting to assess which characteristics affect the variability of the time between the first infection and the moment where an alert should be raised (e.g. when mortality increases above a certain threshold) on the one hand and of the time lag between a theoretical detection and the actual moment where a suspicion was reported on the other. This question falls outside the scope of our study, and addressing it would require more detailed data.

Estimates of the date of first infection in a poultry flock could help contact tracing, by enabling official veterinarians to focus on the relevant time window. We suggest, on the one hand, that backward tracing (which aims at identifying the origin of the outbreak) should be focused on the 95% CrI of the time of first infection, which represents the most likely time window where contact with the source case happened. On the other hand, forward tracing (which aims at identifying secondary cases) should be focused on the most likely time window where infectious contacts to secondary cases are possible. As the latent period could be quite short (see electronic supplementary material) and to avoid missing potential contacts, we made the conservative assumption that infectious contacts could happen from the time of first infection, neglecting the latent period. Therefore, we suggest that forward tracing should be focused from the lower bound of the 95% CrI of the time of first infection up to the day when the flock is culled or successfully isolated.

Our results suggest that the time window used for contact tracing, currently fixed to 21 days [17], could be shortened or extended depending on the flock. For instance, for flock FRA-2021-3 (figure 3) whose estimated time of first infection was 3.5 (95% CrI: 1.8, 6.2) days prior to the last observation, using a 21-day window for contact tracing would mean that a lot of effort and resources are used to trace contacts that happened before virus introduction and are therefore not really relevant. In this case, focusing on, e.g. a 7-day window could have been sufficient, meaning that resources could have been saved for other control or surveillance purposes. On the other hand, for flock FRA-2016-14 with an estimate of 19.9 days (95% CrI: 11.9, 31.3), using a 21-day window means that epidemiological links, potentially including the potential source of infection, could have been missed. In that case, a longer time window, e.g. 32 days, would maximize the chances of capturing all relevant contacts with the infected farm.

Therefore, our approach could be used to tailor the optimal time window to each flock. Moreover, it can be used in real time, as our modelling approach with ABC rejection can provide results in a few minutes and is based on easy-to-collect mortality data. Therefore, we created an interactive web application using the R package Shiny [48], that can be used in the field by official veterinarians. The application can be accessed at: https://first-inf.sk8.inrae.fr/.

As seen in our study, our approach was not always successful with all HPAI-infected flocks. However, we were not able to find the characteristics of the flocks for which the estimation procedure failed, and thus to predict in advance when the estimation would fail. Therefore, in the Shiny app, we put several safeguards in place to warn the end user in case the estimation of the time of first infection did not perform well. First, although this was not the case in our dataset, we displayed a warning when the overlap between prior and posterior was very high (e.g. above 0.80), which would indicate that the data were not informative. Second, we also warned the user when the upper bound of the 95% CrI was equal to or greater than 0.5 days prior to the last observation, as it systematically meant that the model did not fit well with the observed data in our analyses (figure 3, electronic supplementary material). Finally, a posterior predictive check is also performed within the Shiny app, and a warning is displayed when the observed trend in mortality is not adequately captured by the model simulations. In all these situations, the end user is invited to discard the results of the model and to stick with the recommended 21-day time window.

Indeed, our results suggest, in accordance with previous studies [20,23], that in the absence of appropriate data or accurate estimates of the time of first infection, 21 days are generally an appropriate time window for contact tracing, with a few exceptions (figure 3; electronic supplementary materiall). However, when daily mortality data are available and our approach is able to provide estimates of the time of first infection, we recommend using the flock-specific time window to optimize the efficiency of contact tracing.

For the time of first infection, overlaps between prior–posterior pairs indicated that the data supplied information about this parameter. Variations in overlap values between flocks were in part due to the width of the priors, which were narrower for flocks recently introduced in the poultry house (table 1; electronic supplementary material). Indeed, the narrower the prior, the higher the overlap (correlation coefficient: 0.43, *p* < 0*.*001), indicating that a wider prior was more easily restricted relative to the prior with the available data. Variations also reflected the uncertainty contained in the data for each flock. Therefore, although some overlap values were relatively high (e.g. 0.58 for FRA-2016-14) and the corresponding 95% credible intervals wide, this was valuable given our objective of not providing overconfident results and therefore overly constrained time windows for contact tracing.

Unfortunately, our method was not able to satisfactorily estimate other transmission parameters, such as the transmission rate or the probability of dying of disease, as reflected by the very high values (0.60 or above) of the overlap between prior and posterior distributions for the majority of flocks (electronic supplementary material). This could either mean that the true values of these parameters were different from the prior distributions, but that the data were not sufficiently informative for these parameters; or that the true values of these parameters were close to the prior distributions, which were informed by current knowledge on HPAI viruses (table 1 and electronic supplementary material). Both hypotheses are possible, although it was not possible to distinguish between the two. Therefore, estimated values for parameters with high overlaps between prior–posterior pairs (i.e. other than the time of first infection and the natural mortality rate) have to be interpreted with caution. Consequently, although a secondary initial objective of our study was to analyse the characteristics (e.g. poultry species, production system, virus subtype) that could explain the observed variability between estimates of transmission parameters, it was not possible to do so given that posterior distributions for these parameters were mostly driven by prior distributions. Despite close prior and posterior distributions for most flocks, we chose to keep these parameters in the estimation algorithm for two reasons: (i) to reflect the uncertainty around these parameters and the potential variability due to, e.g. host species or virus strain and (ii) to keep a flexible-enough approach to adapt to various flocks, including a few for which the data were actually informative on some transmission parameters (electronic supplementary material). Further work is needed to determine which HPAI transmission parameters can be accurately estimated using mechanistic modelling and mortality data, and how weakly identifiable parameters might influence the estimation of other parameters such as the time of first infection.

Priors were built based on the available literature to reflect the current knowledge on the model parameters. These priors covered a wide range of possible values, reflecting uncertainty but also variability as the data came from a wide variety of HPAI virus strains and epidemiological settings. As such, although the epidemiology of HPAI viruses is constantly changing, especially for HPAI H5Nx viruses in recent years [49], we believe that the priors are adequate for both current and future strains. Moreover, if new knowledge emerged showing that new strains have substantially different characteristics, prior distributions should then be updated accordingly. In contrast, some priors were species specific, namely the average duration of the infectious period and the probability of dying of infection (table 1 and electronic supplementary material). Therefore, our approach has not been tested for other poultry species, such as geese, for which we have no available data. Our approach could be extended to other poultry species, provided that sufficient data become available.

Another limitation is that our approach is restricted to infected poultry flocks displaying abnormal mortality, and cannot be applied to infected flocks that are detected early before any significant increase in mortality (i.e. based on regular diagnostic tests or on abnormal clinical signs). A perspective of our work would therefore be to adapt the model to allow the estimation of the time of first infection based on other types of data that could be easily available. For instance, virological tests of cloacal or tracheal swabs are routinely used to confirm suspicion of HPAI infections, and results of these tests could be used to infer the transmission dynamics (provided that these results are available for individual birds, as swabs are usually pooled prior to testing). Another example of potentially usable data is egg production, e.g. for breeder flocks [28].

Unfortunately, our model is not yet adapted to vaccinated flocks. Therefore, it can no longer be used for vaccinated duck flocks in France, where vaccination started in October 2023 [50]. Moreover, the use of vaccination is expected to reduce mortality due to HPAI, making use of daily mortality data inadequate to estimate the time of first infection. Therefore, alternative models and data should be developed in the future for use in vaccinated poultry flocks.

To conclude, we were able to retrospectively estimate the time of first infection for most flocks in our large dataset of 63 HPAI-infected flocks from France and The Netherlands. Based on these encouraging results, we built a Shiny app that can be used by veterinary services in real time during HPAI epidemics to obtain flock-specific estimates of the time of first infection. In turn, these estimates can be used to tailor the time window used for contact tracing, which is expected to improve the efficiency of this method and the accompanying control measures. This should contribute to improving the overall management of current and future HPAI epidemics, which are more and more frequent and difficult to control given the rapidly mutating nature of the virus.

## Data Availability

All the scripts and data that were used for the analyses are available at [51]. The Shiny app can be accessed at [52]. Supplementary material is available online [53].

## References

[1] Tildesley MJ, Bessell PR, Keeling MJ, Woolhouse MEJ. 2009 The role of pre-emptive culling in the control of foot-and-mouth disease. Proc. R. Soc. B **276**, 3239–3248. (10.1098/rspb.2009.0427) PMC2817163 19570791

[2] Pandey A, Atkins KE, Medlock J, Wenzel N, Townsend JP, Childs JE, Nyenswah TG, Ndeffo-Mbah ML, Galvani AP. 2014 Strategies for containing Ebola in West Africa. Science **346**, 991–995. (10.1126/science.1260612) 25414312 PMC4316831

[3] Vazquez-Prokopec GM, Montgomery BL, Horne P, Clennon JA, Ritchie SA. 2017 Combining contact tracing with targeted indoor residual spraying significantly reduces dengue transmission. Sci. Adv. **3**, e1602024. (10.1126/sciadv.1602024) 28232955 PMC5315446

[4] Woolhouse ME *et al*. 1997 Heterogeneities in the transmission of infectious agents: implications for the design of control programs. Proc. Natl Acad. Sci. USA **94**, 338–342. (10.1073/pnas.94.1.338) 8990210 PMC19338

[5] Galvani AP, May RM. 2005 Epidemiology: dimensions of superspreading. Nature **438**, 293–295. (10.1038/438293a) 16292292 PMC7095140

[6] Lloyd-Smith JO, Schreiber SJ, Kopp PE, Getz WM. 2005 Superspreading and the effect of individual variation on disease emergence. Nature **438**, 355–359. (10.1038/nature04153) 16292310 PMC7094981

[7] Matthews L *et al*. 2006 Heterogeneous shedding of Escherichia coli O157 in cattle and its implications for control. Proc. Natl Acad. Sci. USA **103**, 547–552. (10.1073/pnas.0503776103) 16407143 PMC1325964

[8] Dhillon RS, Srikrishna D. 2018 When is contact tracing not enough to stop an outbreak? Lancet Infect. Dis. **18**, 1302–1304. (10.1016/S1473-3099(18)30656-X) 30507446

[9] Eames KTD, Keeling MJ. 2003 Contact tracing and disease control. Proc. R. Soc. Lond. B **270**, 2565–2571. (10.1098/rspb.2003.2554) PMC1691540 14728778

[10] Fraser C, Riley S, Anderson RM, Ferguson NM. 2004 Factors that make an infectious disease outbreak controllable. Proc. Natl Acad. Sci. USA **101**, 6146–6151. (10.1073/pnas.0307506101) 15071187 PMC395937

[11] Klinkenberg D, Fraser C, Heesterbeek H. 2006 The effectiveness of contact tracing in emerging epidemics. PLoS One **1**, e12. (10.1371/journal.pone.0000012) 17183638 PMC1762362

[12] Lipsitch M *et al*. 2003 Transmission dynamics and control of severe acute respiratory syndrome. Science **300**, 1966–1970. (10.1126/science.1086616) 12766207 PMC2760158

[13] Ferretti L, Wymant C, Kendall M, Zhao L, Nurtay A, Abeler-Dörner L, Parker M, Bonsall D, Fraser C. 2020 Quantifying SARS-CoV-2 transmission suggests epidemic control with digital contact tracing. Science **368**, eabb6936. (10.1126/science.abb6936) 32234805 PMC7164555

[14] Ferguson NM, Donnelly CA, Anderson RM. 2001 Transmission intensity and impact of control policies on the foot and mouth epidemic in Great Britain. Nature **413**, 542–548. (10.1038/35097116) 11586365

[15] Elbers ARW, Moser H, Ekker HM, Crauwels PAA, Stegeman JA, Smak JA, Pluimers FH. 2001 Tracing systems used during the epidemic of classical swine fever in The Netherlands, 1997–1998. Rev. Sci. Tech. Off. Int. Epizoot. **20**, 614–629. (10.20506/rst.20.2.1296) 11548531

[16] Fetzer T, Graeber T. 2021 Measuring the scientific effectiveness of contact tracing: evidence from a natural experiment. Proc. Natl Acad. Sci. USA **118**, e2100814118. (10.1073/pnas.2100814118) 34385318 PMC8380024

[17] European Commission. 2016 Regulation (EU) 2016/429 of the European Parliament and of the Council of 9 March 2016 on transmissible animal diseases and amending and repealing certain acts in the area of animal health (Animal Health Law). Off. J. Eur. Union **L 84**, 1–208.

[18] European Commission. 2020 Commission delegated regulation (EU) 2020/687 of 17 December 2019 supplementing regulation (EU) 2016/429 of the European Parliament and the Council, as regards rules for the prevention and control of certain listed diseases. Off. J. Eur. Union **L 174**, 64–139.

[19] EFSA Panel on Animal Health and Welfare (EFSA AHAW Panel). 2021 Scientific opinion on the assessment of the control measures of the category A diseases of Animal Health Law: highly pathogenic avian influenza. EFSA J. **19**, e06372. (10.2903/j.efsa.2021.6372) 33488812 PMC7812451

[20] Hobbelen PHF, Elbers ARW, Werkman M, Koch G, Velkers FC, Stegeman A, Hagenaars TJ. 2020 Estimating the introduction time of highly pathogenic avian influenza into poultry flocks. Sci. Rep. **10**, 12388. (10.1038/s41598-020-68623-w) 32709965 PMC7381656

[21] Vergne T *et al*. 2021 Inferring within‐flock transmission dynamics of highly pathogenic avian influenza H5N8 virus in France, 2020. Transbound. Emerg. Dis. **68**, 3151–3155. (10.1111/tbed.14202) 34170081 PMC9291964

[22] Nezworski J, St Charles KM, Malladi S, Ssematimba A, Bonney PJ, Cardona CJ, Halvorson DA, Culhane MR. 2021 A retrospective study of early vs. late virus detection and depopulation on egg laying chicken farms infected with highly pathogenic avian influenza virus during the 2015 H5N2 outbreak in the United States. Avian Dis. **65**, 474–482. (10.1637/aviandiseases-D-21-00019) 34699146

[23] Hayama Y, Sawai K, Yoshinori M, Yamaguchi E, Yamamoto T. 2022 Estimation of introduction time window of highly pathogenic avian influenza virus into broiler chicken farms during the 2020–2021 winter season outbreak in Japan. Prev. Vet. Med. **208**, 105768. (10.1016/j.prevetmed.2022.105768) 36174447

[24] Guinat C, Porphyre T, Gogin A, Dixon L, Pfeiffer DU, Gubbins S. 2018 Inferring within-herd transmission parameters for African swine fever virus using mortality data from outbreaks in the Russian Federation. Transbound. Emerg. Dis. **65**, e264–e271. (10.1111/tbed.12748) 29120101 PMC5887875

[25] Stegeman A, Elbers ARW, Bouma A, de Smit H, de Jong MCM. 1999 Transmission of classical swine fever virus within herds during the 1997–1998 epidemic in The Netherlands. Prev. Vet. Med. **42**, 201–218. (10.1016/S0167-5877(99)00076-8) 10619156

[26] Gonzales JL, Elbers ARW, van der Goot JA, Bontje D, Koch G, de Wit JJ, Stegeman JA. 2012 Using egg production data to quantify within-flock transmission of low pathogenic avian influenza virus in commercial layer chickens. Prev. Vet. Med. **107**, 253–259. (10.1016/j.prevetmed.2012.06.010) 22819637

[27] Bonney PJ, Malladi S, Ssematimba A, Spackman E, Torchetti MK, Culhane M, Cardona CJ. 2021 Estimating epidemiological parameters using diagnostic testing data from low pathogenicity avian influenza infected Turkey houses. Sci. Rep. **11**, 1602. (10.1038/s41598-021-81254-z) 33452377 PMC7810853

[28] Elbers ARW, Gonzales JL. 2021 Mortality levels and production indicators for suspicion of highly pathogenic avian influenza virus infection in commercially farmed ducks. Pathogens **10**, 1498. (10.3390/pathogens10111498) 34832653 PMC8620262

[29] Gonzales JL, Hobbelen PHF, van der Spek AN, Vries EP, Elbers ARW. 2023 Highly pathogenic avian influenza a H5 virus outbreaks in broiler farms in The Netherlands—clinical signs, transmission and identification of reporting thresholds. bioRxiv 2023.01.05.522008. (10.1101/2023.01.05.522008)

[30] Gonzales JL, Elbers ARW. 2018 Effective thresholds for reporting suspicions and improve early detection of avian influenza outbreaks in layer chickens. Sci. Rep. **8**, 8533. (10.1038/s41598-018-26954-9) 29867092 PMC5986775

[31] DGAl. 2016 Arrêté du 16 mars 2016 relatif aux niveaux du risque épizootique en raison de l’infection de l’avifaune par un virus de l’influenza aviaire hautement pathogène et aux dispositifs associés de surveillance et de prévention chez les volailles et autres oiseaux captifs. J. Off. Rep. Fr. **0076**, 98. https://www.legifrance.gouv.fr/eli/arrete/2016/3/16/AGRG1604341A/jo/textehttps://www.legifrance.gouv.fr/eli/arrete/2016/3/16/AGRG1604341A/jo/texte

[32] Wearing HJ, Rohani P, Keeling MJ. 2005 Appropriate models for the management of infectious diseases. PLoS Med. **2**, e174. (10.1371/journal.pmed.0020174) 16013892 PMC1181873

[33] Toni T, Welch D, Strelkowa N, Ipsen A, Stumpf MPH. 2009 Approximate Bayesian computation scheme for parameter inference and model selection in dynamical systems. J. R. Soc. Interface **6**, 187–202. (10.1098/rsif.2008.0172) 19205079 PMC2658655

[34] Lenormand M, Jabot F, Deffuant G. 2013 Adaptive approximate Bayesian computation for complex models. Comput. Stat. **28**, 2777–2796. (10.1007/s00180-013-0428-3)

[35] Jabot F, Faure T, Dumoulin N. 2013 EasyABC: performing efficient approximate Bayesian computation sampling schemes using R. Methods Ecol. Evol. **4**, 684–687. (10.1111/2041-210X.12050)

[36] Jabot F, Faure T, Dumoulin N, Albert C. 2023 EasyABC: efficient approximate Bayesian computation sampling schemes. R package version 1.5.2. See https://CRAN.R-project.org/package=EasyABC.

[37] Kirkeby C, Ward MP. 2022 A review of estimated transmission parameters for the spread of avian influenza viruses. Transbound. Emerg. Dis. **69**, 3238–3246. (10.1111/tbed.14675) 35959696 PMC10088015

[38] Lambert S, Bauzile B, Mugnier A, Durand B, Vergne T, Paul MC. 2023 A systematic review of mechanistic models used to study avian influenza virus transmission and control. Vet. Res. **54**, 96. (10.1186/s13567-023-01219-0) 37853425 PMC10585835

[39] Spekreijse D, Bouma A, Stegeman JA, Koch G, de Jong MCM. 2011 The effect of inoculation dose of a highly pathogenic avian influenza virus strain H5N1 on the infectiousness of chickens. Vet. Microbiol. **147**, 59–66. (10.1016/j.vetmic.2010.06.012) 20619974

[40] Germeraad EA, Sanders P, Hagenaars TJ, de Jong MCM, Beerens N, Gonzales JL. 2019 Virus shedding of avian influenza in poultry: a systematic review and meta-analysis. Viruses **11**, 812. (10.3390/v11090812) 31480744 PMC6784017

[41] Grund C *et al*. 2018 A novel European H5N8 influenza a virus has increased virulence in ducks but low zoonotic potential. Emerg. Microbes Infect. **7**, 1–14. (10.1038/s41426-018-0130-1) 30026505 PMC6053424

[42] Beerens N, Germeraad EA, Venema S, Verheij E, Pritz-Verschuren SBE, Gonzales JL. 2021 Comparative pathogenicity and environmental transmission of recent highly pathogenic avian influenza H5 viruses. Emerg. Microbes Infect. **10**, 97–108. (10.1080/22221751.2020.1868274) 33350337 PMC7832006

[43] Bessière P *et al*. 2022 Opposite outcomes of the within-host competition between high- and low-pathogenic H5N8 avian influenza viruses in chickens compared to ducks. J. Virol. **96**, e0136621. (10.1128/JVI.01366-21) 34613804 PMC8754203

[44] Gimenez O, Morgan BJT, Brooks SP. 2009 Weak identifiability in models for mark–recapture–recovery data. In Modeling demographic processes in marked populations (eds DL Thomson, EG Cooch, MJ Conroy), pp. 1055–1067. Boston, MA: Springer. (10.1007/978-0-387-78151-8_48)

[45] R Core Team. 2023 R: a language and environment for statistical computing. Vienna, Austria: R Foundation for Statistical Computing. See http://www.R-project.org/.

[46] Tavaré S, Balding DJ, Griffiths RC, Donnelly P. 1997 Inferring coalescence times from dna sequence data. Genetics **145**, 505–518. (10.1093/genetics/145.2.505) 9071603 PMC1207814

[47] Pritchard JK, Seielstad MT, Perez-Lezaun A, Feldman MW. 1999 Population growth of human Y chromosomes: a study of Y chromosome microsatellites. Mol. Biol. Evol. **16**, 1791–1798. (10.1093/oxfordjournals.molbev.a026091) 10605120

[48] Chang W *et al*. 2023 Shiny: web application framework for R. R package version 1.8.1.1. See https://CRAN.R-project.org/package=shiny.

[49] Xie R *et al*. 2023 The episodic resurgence of highly pathogenic avian influenza H5 virus. Nature **622**, 810–817. (10.1038/s41586-023-06631-2) 37853121

[50] DGAl. 2023 Avian influenza: the French vaccination plan. Ministère de l’Agriculture, de la Souveraineté alimentaire et de la Forêt. See https://agriculture.gouv.fr/tout-ce-quil-faut-savoir-sur-le-plan-daction-vaccination-iahp-en-france (accessed 22 December 2023).

[51] Lambert S. 2024 Data from: Optimising contact tracing for avian influenza in poultry flocks - scripts and data. IHAP. (10.57745/6CQC0Z) PMC11706645 39772734

[52] Lambert S, Fourtune L, Hobbelen PHF, Baca J, Gonzales JL, Elbers ARW, Vergne T. FIRST-INF: Estimating the date of first infection by highly pathogenic avian influenza in a poultry flock. See https://first-inf.sk8.inrae.fr/.

[53] Lambert S, Fourtune L, Hobbelen PHF, Baca J, Gonzales JL, Elbers ARW, Vergne T. 2024 Supplementary material from: Optimising contact tracing for avian influenza in poultry flocks. Figshare. (10.6084/m9.figshare.c.7571808) PMC11706645 39772734

